# Association of genetic variants with hypertension in a longitudinal population-based genetic epidemiological study

**DOI:** 10.3892/ijmm.2015.2151

**Published:** 2015-03-20

**Authors:** YOSHIJI YAMADA, KOTA MATSUI, ICHIRO TAKEUCHI, MITSUTOSHI OGURI, TETSUO FUJIMAKI

**Affiliations:** 1 Department of Human Functional Genomics, Life Science Research Center, Mie University, Tsu, Mie 514-8507, Japan; 2 Core Research for Evolutionary Science and Technology (CREST), Japan Science and Technology Agency, Tokyo 102-0076, Japan; 3 Department of Scientific and Engineering Simulation, Graduate School of Engineering, Nagoya Institute of Technology, Nagoya 466-8555, Japan; 4 Department of Cardiology, Japanese Red Cross Nagoya First Hospital, Nagoya 453-8511, Japan; 5 Department of Cardiovascular Medicine, Inabe General Hospital, Inabe, Mie 511-0428, Japan

**Keywords:** hypertension, genetics, polymorphism, genetic epidemiology, longitudinal study

## Abstract

We previously identified 9 genes and chromosomal region 3q28 as susceptibility loci for Japanese patients with myocardial infarction, ischemic stroke, or chronic kidney disease by genome-wide or candidate gene association studies. In the present study, we investigated the possible association of 13 single nucleotide polymorphisms (SNPs) at these 10 loci with the prevalence of hypertension or their association with blood pressure (BP) in community-dwelling individuals in Japan. The study subjects comprised 6,027 individuals (2,250 subjects with essential hypertension, 3,777 controls) who were recruited into the Inabe Health and Longevity Study, a longitudinal genetic epidemiological study on atherosclerotic, cardiovascular and metabolic diseases. The subjects were recruited from individuals who visited the Health Care Center of Inabe General Hospital for an annual health checkup, and they are followed up each year (mean follow-up period, 5 years). Longitudinal analysis with a generalized estimating equation and with adjustment for age, gender, body mass index and smoking status revealed that rs2116519 of family with sequence similarity 78, member B (*FAM78B*; P=0.0266), rs6929846 of butyrophilin, subfamily 2, member A1 (*BTN2A1*; P= 0.0013), rs146021107 of pancreatic and duodenal homeobox 1 (*PDX1*; P=0.0031) and rs1671021 of lethal giant larvae homolog 2 (*Drosophila*) (*LLGL2*; P=0.0372) were significantly (P<0.05) associated with the prevalence of hypertension. Longitudinal analysis with a generalized linear mixed-effect model and with adjustment for age, gender, body mass index and smoking status among individuals not taking anti-hypertensive medication revealed that rs6929846 of *BTN2A1* was significantly associated with systolic (P=0.0017), diastolic (P=0.0008) and mean (P=0.0005) BP, and that rs2116519 of *FAM78B*, rs146021107 of *PDX1* and rs1671021 of *LLGL2* were significantly associated with diastolic (P=0.0495), systolic (P=0.0132), and both diastolic (P=0.0468) and mean (0.0471) BP, respectively. *BTN2A1* may thus be a susceptibility gene for hypertension.

## Introduction

Hypertension is a complex multifactorial disorder that is thought to result from an interaction between an individual’s genetic background and various lifestyle and environmental factors (1). The genetic influence on blood pressure (BP) variability has been estimated at 30–60% for a given individual (2), and the genetic heritability of hypertension estimated at 30% (3). Given that hypertension is a major risk factor for coronary artery disease, ischemic and hemorrhagic stroke, as well as chronic kidney disease (4–6), the personalized prevention of hypertension is an important public health goal.

Genome-wide association studies have identified various loci and genes associated with BP or to a predisposition to hypertension in Caucasian populations (7–11) or African Americans (12). Although the genes for adducin 2 (13) and ATPase, Ca^2+^ transporting, plasma membrane 1 (14) have been shown to be susceptibility loci for hypertension in Japanese individuals, the genes that confer susceptibility to this condition in Japanese individuals remain to be identified definitively.

We have previously identified 9 genes and chromosomal region 3q28 as susceptibility loci for myocardial infarction, ischemic stroke, or chronic kidney disease in Japanese individuals by genome-wide (15–17) or candidate gene (18–20) association studies. Given that hypertension is an important risk factor for these conditions (4–6), we hypothesized that certain single nucleotide polymorphisms (SNPs) at these 10 loci may contribute to their genetic susceptibility by affecting the susceptibility to hypertension. Therefore, the purpose of the present study was to examine the possible association of 13 SNPs at these 10 loci with the prevalence of essential hypertension or their association with BP in community-dwelling Japanese individuals.

## Materials and methods

### Study population

Study subjects comprised 6,027 community-dwelling individuals (2,250 subjects with essential hypertension and 3,777 controls) who were recruited to a population-based cohort study in Inabe City (Inabe Health and Longevity Study), Mie Prefecture, Japan. The Inabe Health and Longevity Study is a longitudinal genetic epidemiological study of atherosclerotic, cardiovascular and metabolic diseases (21–26). The subjects were recruited from individuals who visited the Health Care Center of Inabe General Hospital for an annual health checkup, and they are followed up each year. A total of 6,027 individuals was registered between March 2010 and September 2012, and genomic DNA was extracted from the venous blood cells of these subjects and stored in the genomic DNA bank of the Research Center for Genomic Medicine at Mie University. For all the participants, medical examination data obtained from April 2003 to March 2014 (11 years) were entered into a database. If individuals had a medical checkup 2 or more times per year, data from one time point for each year were entered, so that each subject had one set of health data for each year they had attended the clinic. All participants thus had undergone 1–11 medical examinations, and the average follow-up period was 5 years.

Subjects with hypertension either had a systolic BP of ≥140 mmHg or a diastolic BP of ≥90 mmHg (or both) or had taken anti-hypertensive medication. The control individuals had a systolic BP of <140 mmHg and a diastolic BP of <90 mmHg, as well as no history of hypertension or of taking any anti-hypertensive medication. BP was measured at least twice with the subjects having rested in the sitting position for >5 min; the measurements were taken by a skilled physician or nurse according to the guidelines of the American Heart Association (27). The study protocol complied with the Declaration of Helsinki and was approved by the Committees on the Ethics of Human Research of Mie University Graduate School of Medicine and Inabe General Hospital. Written informed consent was obtained from all subjects prior to enrollment in the study.

### Selection and genotyping of polymorphisms

The 13 SNPs examined in the present study (Table I) were selected from our previous genome-wide (15–17) or candidate gene (18–20) association studies. Wild-type (ancestral) and variant alleles of the SNPs were determined from the dbSNP database (National Center for Biotechnology Information, Bethesda, MD, USA) (http://www.ncbi.nlm.nih.gov/SNP).

Venous blood (5 ml) was collected into tubes containing 50 mmol/l ethylenediaminetetraacetic acid (disodium salt), and peripheral blood leukocytes were isolated and genomic DNA was extracted from these cells with the use of a DNA extraction kit (SMITEST EX-R&D; Medical and Biological Laboratories, Nagoya, Japan). The genotypes of the 13 SNPs were determined at G&G Science Co., Ltd. (Fukushima, Japan) by a method that combines the polymerase chain reaction and sequence-specific oligonucleotide probes with suspension array technology (Luminex, Austin, TX, USA). The primers, probes and other conditions for the genotyping of the SNPs examined in the present study are shown in Table II. Detailed genotyping methodology was as described previously (15,16,28).

### Statistical analysis

Quantitative data were compared between the subjects with hypertension and the controls with the unpaired Student’s t-test. Categorical data were compared with the χ^2^ test. We examined the association of the 13 SNPs with the prevalence of hypertension or their association with systolic, diastolic, or mean BP based on a 5-year longitudinal cohort study. Longitudinal changes in the prevalence of hypertension were compared between 2 groups (the dominant or recessive genetic model) with a generalized estimating equation, as previously described (29) and with adjustment for age, gender, body mass index (BMI) and smoking status. Longitudinal changes in systolic, diastolic, or mean BP in all the individuals or in the individuals not any taking anti-hypertensive medication were compared between 2 groups (the dominant or recessive model) in a generalized linear mixed-effect model, as previously described (30) with adjustment for age, gender, BMI and smoking status. The dominant or recessive model was defined as *AA* vs. *AB* + *BB* or *AA* + *AB* vs. *BB* (*A*, major allele; *B*, minor allele), respectively. Age-related changes in the prevalence of hypertension or in systolic or diastolic BP were estimated with quadratic curves controlling for the observation year. A P-value <0.05 was considered to indicate a statistically significant difference. Statistical analysis was performed using R software version 3-0-2 (the R Project for Statistical Computing) and JMP Genomics version 6.0 (SAS Institute, Cary, NC, USA).

## Results

Characteristics of the 6,027 study subjects (3,352 males, 2,675 females) with regard to all measurements in a 5-year follow-up are shown in Table III. Characteristics of the subjects with hypertension and the controls according to cross-sectional analysis in March 2014 are shown in Table IV. Age, the frequency of the male gender, BMI and the prevalence of smoking were greater in the subjects with hypertension than in the controls.

The association of the 13 SNPs with the prevalence of hypertension was analyzed with a generalized estimating equation and with adjustment for age, gender, BMI and smoking status (Table V). The rs2116519 (C→T) SNP of the family with sequence similarity 78, member B gene (*FAM78B*, recessive model), rs6929846 (T→C) of the butyrophilin, subfamily 2, member A1 gene (*BTN2A1*, dominant model), rs146021107 (G→-) of the pancreatic and duodenal homeobox 1 gene (*PDX1*, dominant model) and rs1671021 (G→A) of the lethal giant larvae homolog 2 gene (*LLGL2*, dominant model) were significantly (P<0.05) associated with the prevalence of hypertension.

**Figure 1:**
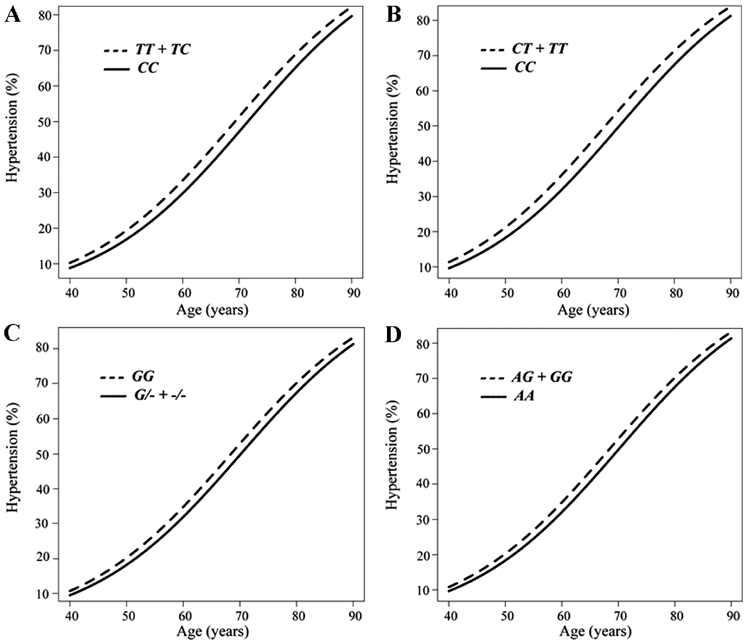
Longitudinal analysis with a generalized estimating equation of the association between the prevalence of hypertension and age according to the genotype for (A) rs2116519 of *FAM78B* (*TT* + *TC* vs. *CC*), (B) rs6929846 of *BTN2A1* (*CC* vs. *CT* + *TT*) (B), (C) rs146021107 of *PDX1* (*GG* vs. *G/*- + -/-), or (D) rs1671021 of *LLGL2* (*AA* vs. *AG* + *GG*).

The association between the prevalence of hypertension and age analyzed longitudinally with a generalized estimating equation according to the SNP genotype is shown in Fig. 1. The prevalence of hypertension was greater in the combined group of subjects with the *TT* or *TC* genotypes of rs2116519 of *FAM78B* than in those with the *CC* genotype from 40 to 90 years of age (Fig. 1A), in the combined group of subjects with the *CT* or *TT* genotypes of rs6929846 of *BTN2A1* than in those with the *CC* genotype (Fig. 1B), in subjects with the *GG* genotype of rs146021107 of *PDX1* than in the combined group of subjects with the *G/*- or -/- genotypes (Fig. 1C), and in the combined group of subjects with the *AG* or *GG* genotypes of rs1671021 of *LLGL2* than in those with the *AA* genotype (Fig. 1D).

Given that 4 SNPs were significantly associated with hypertension, the association of these SNPs with systolic, diastolic, or mean BP in all individuals or individuals not taking any anti-hypertensive medication were analyzed with a generalized linear mixed-effect model, with adjustment for age, gender, BMI and smoking status (Table VI). The rs6929846 polymorphism of *BTN2A1* was significantly associated with systolic, diastolic and mean BP in the dominant model among all individuals or individuals not taking any anti-hypertensive medication, with the *T* allele being associated with an increased BP. The rs146021107 SNP of *PDX1* was significantly associated with systolic BP in the dominant model among all individuals or individuals not taking any anti-hypertensive medication, with the *G* allele being associated with an increased BP. The rs2116519 polymorphism of *FAM78B* was significantly associated with diastolic BP in the recessive model among individuals not taking any anti-hypertensive medication, with the *T* allele being associated with a high BP. The rs1671021 SNP of *LLGL2* was significantly associated with diastolic and mean BP in the dominant model among individuals not taking any anti-hypertensive medication, with the *G* allele being associated with a high BP.

**Figure 2:**
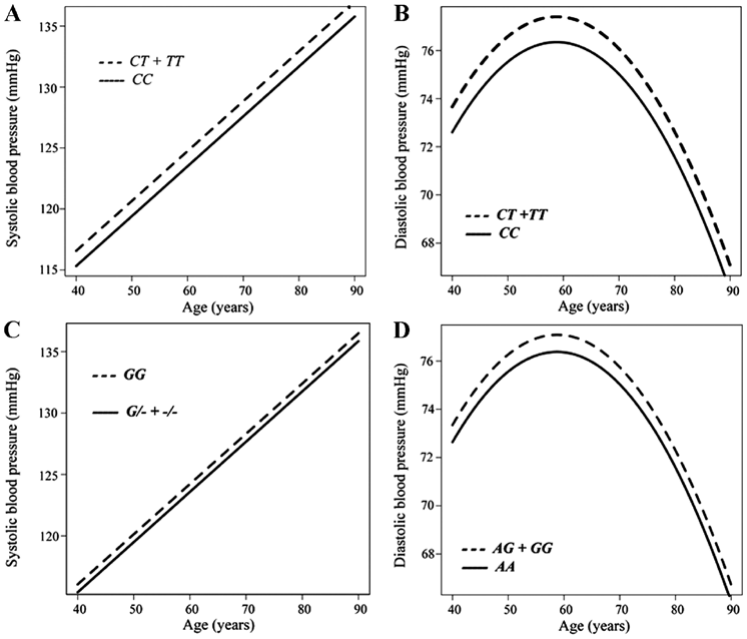
Longitudinal analysis with a generalized linear mixed-effect model of the association between (A) systolic or (B) diastolic blood pressure (BP) and age according to genotype for rs6929846 of *BTN2A1* (*CC* vs. *CT* + *TT*), (C) between systolic BP and age according to genotype for rs146021107 of *PDX1* (*GG* vs. *G/*- + -/-), or (D) between diastolic BP and age according to genotype for rs1671021 of *LLGL2* (*AA* vs. *AG* + *GG*) among individuals not taking any anti-hypertensive medication.

The association between systolic or diastolic BP and age in individuals not taking any anti-hypertensive medication was analyzed longitudinally according to genotype with a generalized linear mixed-effect model (Fig. 2). Systolic (Fig. 2A) and diastolic (Fig. 2B) BP were greater in the combined group of individuals with the *CT* or *TT* genotypes of rs6929846 of *BTN2A1* than in those with the *CC* genotype from 40 to 90 years of age. Systolic BP was greater in subjects with the *GG* genotype of rs146021107 of *PDX1* than in the combined group of individuals with the *G/*- or -/- genotypes (Fig. 2C). Diastolic BP was greater in the combined group of individuals with the *AG* or *GG* genotypes of rs1671021 of *LLGL2* than in those with the *AA* genotype (Fig. 2D).

## Discussion

Given that genetic factors, as well as interactions between multiple genes and environmental factors are important in the development of hypertension (1), the ability to predict the risk of developing hypertension on the basis of genetic variants would be beneficial for the personalized prevention of this condition. In this study, we demonstrated that rs6929846 (T→C) of *BTN2A1* was significantly associated with the prevalence of hypertension and also with systolic, diastolic, and mean BP in community-dwelling Japanese individuals, with the minor *T* allele representing a risk factor for hypertension.

We have previously reported that rs6929846 of *BTN2A1* is significantly associated with hypertension in a cross-sectional study of a different hospital-based population (31). We also observed the association of this polymorphism with hypertension in a previous cross-sectional analysis of the Inabe Health and Longevity Study (26). The results of the present longitudinal population-based study are thus consistent with these previous observations (26,31) and validate the association of rs6929846 of *BTN2A1* with hypertension.

*BTN2A1* is a cell-surface transmembrane glycoprotein and a member of the butyrophilin superfamily of proteins. Many of these proteins regulate immune function, and polymorphisms within the coding sequences of the corresponding genes have been associated with the predisposition to inflammatory diseases (32). We have previously demonstrated that the *T* allele of rs6929846 of *BTN2A1* is associated with an increased risk of developing myocardial infarction and with an increased transcriptional activity of *BTN2A1* (15). The serum concentration of high-sensitivity C-reactive protein was significantly greater in individuals in the combined group of *CT* or *TT* genotypes for this SNP than in those with the *CC* genotype among healthy subjects without neoplastic, infectious, or inflammatory disease (15,33). These observations suggest that the *T* allele of rs6929846 of *BTN2A1* may accelerate inflammatory processes.

Previous studies have suggested that chronic vascular inflammation influences BP and vascular remodeling (34–37). Systolic and diastolic BP, as well as pulse pressure were thus found to be positively associated with the plasma concentration of interleukin-6 in healthy men (34). The plasma concentration of high-sensitivity C-reactive protein was also greater in individuals with hypertension than in the controls, and it was shown to be positively associated with systolic BP and pulse pressure (35). In addition, oxidative stress and vascular inflammation have been shown to influence BP, suggesting that chronic inflammation may play a key role in the pathogenesis of hypertension (36,37). In this study, we demonstrated that rs6929846 of *BTN2A1* was significantly associated with hypertension, with the minor *T* allele representing a risk factor for this condition. The enhancement of chronic inflammation by the *T* allele of rs6929846 may account for its association with hypertension, although the molecular mechanisms underlying the effects of this polymorphism on the development of hypertension remain to be elucidated.

In a previous meta-analysis of cohort studies, a reduction of 10 mmHg in systolic or 5 mmHg in diastolic BP was estimated to result in a 22–25% decrease in the incidence of coronary artery disease and a 36–41% decrease in that of stroke (38). In our longitudinal analysis, systolic, diastolic and mean BP were each increased by 1 mmHg in individuals with the *TT* genotype of rs6929846 of *BTN2A1* compared with those with the *CC* genotype. Such a difference is small at the individual level and may not have practical clinical implications. However, even small increments in BP have important effects on cardiovascular morbidity and mortality at the population level, given the high incidence of coronary artery disease, stroke and chronic kidney disease. The reduction in the mortality rate estimated for each 2-mmHg decrease in systolic BP is 4% for coronary artery disease and 6% for stroke (39). Small differences in average BP at the population level thus result in significant differences in the population mortality rate (39).

In this study, we observed that the SNPs of *PDX1*, *LLGL2* and *FAM78B* were also associated with the prevalence of hypertension, as well as with systolic BP among all individuals and individuals not taking any anti-hypertensive medication (*PDX1*), with diastolic and mean BP among individuals without anti-hypertensive medication (*LLGL2*), or with diastolic BP among individuals without anti-hypertensive medication (*FAM78B*). *FAM78B* is located at 1q24.1, which has previously been suggested to harbor susceptibility loci for hypertension (40) and type 2 diabetes mellitus (41), although the function of the gene remains unclear. *PDX1* is a transcriptional activator at several genes, including those for insulin, somatostatin, glucokinase, islet amyloid polypeptide and glucose transporter type 2 (NCBI Gene). It contributes to the early development of the pancreas and plays an important role in the glucose-dependent regulation of insulin gene expression (42). A rare frameshift variant of *PDX1* was previously found to associated with type 2 diabetes mellitus (43). We have previously demonstrated that rs146021107 of *PDX1* is significantly associated with myocardial infarction (18,20), although, to the best of our knowledge, the association of *PDX1* polymorphisms with hypertension has not yet been reported. *LLGL2* plays a role in asymmetric cell division, the establishment of epithelial cell polarity and cell migration (44,45). We have previously demonstrated that rs1671021 of *LLGL2* is associated with ischemic stroke (16), although, to the best of our knowledge, variants of *LLGL2* have not yet been associated with hypertension.

The present study had certain limitations: i) given that the study subjects comprised only Japanese individuals, further studies are required on other ethnic groups. ii) It is possible that rs6929846 of *BTN2A1* is in linkage disequilibrium with other polymorphisms in *BTN2A1* or in nearby genes that are actually responsible for the development of hypertension. iii) The functional relevance of rs6929846 of *BTN2A1* to the pathogenesis of hypertension remains unclear.

In conclusion, the present results suggest that *BTN2A1* is a susceptibility gene for essential hypertension in Japanese individuals. The determination of the genotype for rs6929846 may prove informative for the assessment of the genetic risk for hypertension in such individuals.

## Figures and Tables

**Table I:** The 13 SNPs examined in the present study.

Chromosomal locus	Gene	dbSNP (NCBI)	Nucleotide substitution	Minor allele^a^
1q24.1	*FAM78B*	rs2116519	C→T	C
3q28	Non-gene region	rs9846911	A→G	G
4q25	*ALPK1*	rs2074379	G→A (Met732Ile)	G
4q25	*ALPK1*	rs2074380	G→A (Gly870Ser)	A
4q25	*ALPK1*	rs2074381	A→G (Asn916Asp)	G
4q25	*ALPK1*	rs2074388	G→A (Gly565Asp)	G
6p22.1	*BTN2A1*	rs6929846	T→C	T
6q27	*THBS2*	rs8089	T→G	G
13q12.1	*PDX1*	rs146021107	G→- (deletion)	–
13q34	*F7*	rs6046	G→A (Arg353Gln)	A
17q25.1	*LLGL2*	rs1671021	G→A (Leu479Phe)	G
19p13.2	*ILF3*	rs2569512	G→A	A
22q13.3	*CELSR1*	rs6007897	C→T (Ala2268Thr)	C

a The minor allele in Japanese individuals was determined by the allele frequency of HapMap-JPT in dbSNP. SNPs, single nucleotide polymorphisms.

**Table II:** Primers, probes and other conditions for the genotyping of the 13 SNPs examined in the present study.

Gene or locus	SNP	dbSNP	Sense primer (5′→3′)	Antisense primer (5′→3′)	Probe 1 (5′→3′)	Probe 2 (5′→3′)	Annealing	(°C) Cycles
*FAM78B*	C→T	rs2116519	CCTGCACTGCTCTAGCTACTTC	GATCCCAATTTCAACTGTGAGATC	TCATTCCGGTCTCAGCCGCT	CCCTCATTCCGGTTTCAGCC	60	50
3q28	A→G	rs9846911	AGTTGTGTGCCAGATTCTCCAG	TCTTCACTGAGACCTTGGGAAG	TCTCCTCTTTCAATAACAAATCTTC	AAAGTCTCCTCTTTCAGTAACAAAT	60	50
*ALPK1*	G→A	rs2074379	TCTGCTTCTTGGTCTTCTGATTC	AGTTGGTTTCTGGAAACTCAACAA	GAAGGATGTGTGCCTATATTCTT	GATGTGTGCCCATATTCTTGGG	60	50
*ALPK1*	G→A	rs2074380	CTCCACAGTGGATGAGGAGG	CTTACAGAGGAATTGGGGGTC	ACAAATGGGCACAGCTCTCATA	TATGAGAGCCGTGCCCATTTGT	60	50
*ALPK1*	A→G	rs2074381	AGGACTGCACTACCACAGAGG	TGATTTCAGCCACCACACTGAG	ATCAGCCTGGAAACATGCTAAAC	AGTTTAGCATGTCTCCAGGCTG	60	50
*ALPK1*	G→A	rs2074388	TGTGGAGACTGAGACTGAGCC	TTGCTCCAAGCACTGGAAGTC	ACTACAGCAATGATGAGGGAGC	GCTCCCTCACCATTGCTGTAG	60	50
*BTN2A1*	T→C	rs6929846	CCAAACATGGCGACCTAGGAGA	ATCTGCCCAGGGGCACAGGC	TTTGGGAAGGTTTGCGTCTAG	TTTGGGAAGGTTTGTGTCTAGT	60	50
*THBS2*	T→G	rs8089	AACCCAAGTGCCTTCAGAGGAT	CTCCACATAAAGTCTCATATATCAC	GATGTTCATCTCTGAGTTCCA	GATGTTCATCTCTGCGTTCCA	60	50
*PDX1*	G→-	rs146021107	TGGCTGTGGGTTCCCTCTGAG	GATTTGGCACTGTGTGGCGTTC	CGAGCAGGGGTGGCGCC	GGCGCCACCCTGCTCGCT	60	50
*F7*	G→A	rs6046	CGGCTACTCGGATGGCAGCA	CCAAAGTGGCCCACGGTTGC	TACCACGTGCCCCGGTAGTG	GCCACCCACTACCAGGGCA	60	50
*LLGL2*	G→A	rs1671021	GCTCCTGGCCTCACCTTGCG	GCTGCTCTACAAACTCAGCACTG	CTGGGCACTGAAGTTCTCGTT	CCAACGAGAACCTCAGTGCC	60	50
*ILF3*	G→A	rs2569512	ACCACCTCAACTGCAAGCTGAA	GGAATGATCCCTCTGGGAAGGT	GTGCAACTGCCAAAAACTGGT	GTGCAACTGCCAAGAACTGG	60	50
*CELSR1*	C→T	rs6007897	GGAGACGGAGGACTCCAGCTC	CTTGCTGTCGACATCTTTGACAAG	TCTTCATGGATGGCGTCGAAT	TCTTCATGGATGGTGTCGAATC	60	50

SNPs, single nucleotide polymorphisms.

**Table III:** Characteristics of the study subjects: analysis of all measurements in a 5-year follow-up.

Parameter	Male^a^	Female^a^	All^a^
No. of subjects	3352	2675	6027
Age (years)	52.5±12.5 (15,959)	52.5±11.9 (12,572)	52.5±12.2 (28,531)
Height (cm)	168.4±6.6 (15,550)	155.2±5.9 (12,373)	162.6±9.1 (27,923)
Weight (kg)	67.0±11.0 (15,548)	53.5±8.2 (12,373)	61.0±12.0 (27,921)
Body mass index (kg/m^2^)	23.6±3.3 (15,548)	22.2±3.2 (12,373)	23.0±3.3 (27,921)
Waist circumference (cm)	83.2±8.7 (11,817)	77.8±9.0 (9,541)	80.8±9.2 (21,358)
Alcohol concumption (%)	67.4 (15,959)	26.4 (12,572)	49.3 (28,531)
Current or former smoker (%)	65.0 (15,959)	8.5 (12,572)	40.1 (28,531)
Systolic blood pressure (mmHg)	122±16 (15,541)	119±16 (12,370)	121±16 (27,911)
Diastolic blood pressure (mmHg)	77±12 (15,541)	71±11 (12,370)	75±12 (27,911)
Mean blood pressure (mmHg)	92±13 (15,541)	87±12 (12,370)	90±13 (27,911)
Ocular tension (right, mmHg)	14.0±3.0 (6,132)	13.4±2.8 (4,886)	13.7±3.0 (11,018)
Functional vital capacity (l)	3.53±0.66 (6,173)	2.55±0.47 (4,865)	3.10±0.76 (11,038)
FEV1% (%)	82.3±7.1 (6,168)	84.8±6.7 (4,865)	83.4±7.0 (11,033)
Serum albumin (g/l)	44.5±2.9 (10,332)	44.1±2.7 (8,510)	44.3±2.8 (18,842)
Serum total cholesterol (mmol/l)	5.15±0.88 (15,121)	5.31±0.88 (11,887)	5.22±0.89 (27,008)
Serum triglyceride (mmol/l)	1.46±1.06 (15,639)	1.01±0.58 (12,401)	1.26±0.91 (28,040)
Serum HDL-cholesterol (mmol/l)	1.47±0.39 (15,627)	1.78±0.42 (12,378)	1.61±0.43 (28,005)
Serum LDL-cholesterol (mmol/l)	3.19±0.81 (14,997)	3.18±0.79 (11,836)	3.18±0.80 (26,833)
Fasting plasma glucose (mmol/l)	5.82±1.27 (15,685)	5.39±0.93 (12,395)	5.63±1.15 (28,080)
Blood hemoglobin A1c (%)	5.78±0.74 (10,849)	5.64±0.54 (10,169)	5.71±0.66 (21,018)
Blood urea nitrogen (mmol/l)	5.61±2.86 (8,889)	5.07±2.28 (8,162)	5.36±2.61 (17,051)
Serum creatinine (*μ*mol/l)	88.3±116.2 (14,545)	63.1±82.5 (11,225)	77.3±103.6 (25,770)
eGFR (ml/min/1.73 m^−2^)	77.2±18.0 (14,545)	80.3±17.5 (11,225)	78.5±17.9 (25,770)
Serum uric acid (*μ*mol/l)	372±79 (14,368)	273±62 (10,900)	329±87 (25,268)
Serum C-reactive protein (*μ*g/l)	1573±6428 (5,793)	1207±4107 (4,938)	1405±5486 (10,731)
White blood cells (10^3^/*μ*l)	5.94±1.73 (12,521)	5.03±1.45 (9,419)	5.55±1.68 (21,940)
Red blood cells (10^4^*/μl*)	461±46 (12,651)	415±36 (9,500)	441±47 (22,151)
Hemoglobin (g/l)	147±13 (12,651)	127±13 (9,501)	139±16 (22,152)
Hematocrit (%)	43.3±3.7 (12,642)	37.5±3.4 (9,497)	40.8±4.6 (22,139)
Platelets (104/*μ*l)	23.1±5.5 (12,473)	23.8±6.2 (9,398)	23.4±5.8 (21,871)

a Values in parentheses indicate the numbers of measurements taken. Quantitative data are the means ± SD. FEV1%, forced expiratory volume in 1 sec percentage; HDL, high density lipoprotein; LDL, low density lipoprotein; eGFR, estimated glomerular filtration rate (ml/min/1.73 m^−2^) = 194 × [age (years)]^−0.287^ × [serum creatinine (mg/dl)]^−1.094^ × [0.739 if female].

**Table IV:** Characteristics of subjects with hypertension and controls: cross-sectional analysis in March 2014.

Parameter	Subjects with hypertension^a^	Controls^a^	P-value
No. of subjects	2250	3777	
Age (years)	61.1±10.7 (2,250)	50.1±12.4 (3,777)	<0.0001
Gender (male/female, %)	62.6/37.4	51.5/48.5	<0.0001
Height (cm)	161.3±9.4 (2,207)	163.2±9.0 (3,747)	<0.0001
Weight (kg)	63.1±12.7 (2,205)	59.7±11.6 (3,747)	<0.0001
Body mass index (kg/m^2^)	24.1±3.6 (2,205)	22.3±3.1 (3,747)	<0.0001
Waist circumference (cm)	84.0±9.5 (1,986)	78.5±8.5 (3,619)	<0.0001
Alcohol consumption (%)	52.0 (2,250)	46.0 (3,777)	<0.0001
Current or former smoker (%)	47.7 (2,250)	44.5 (3,777)	0.0147
Systolic blood pressure (mmHg)	133±15 (2,200)	113±11 (3,745)	<0.0001
Diastolic blood pressure (mmHg)	83±12 (2,200)	70±10 (3,745)	<0.0001
Mean blood pressure (mmHg)	99±12 (2,200)	84±9 (3,745)	<0.0001
Ocular tension (right, mmHg)	13.9±3.0 (722)	13.3±2.9 (1,339)	<0.0001
Functional vital capacity (l)	3.12±0.80 (768)	3.39±0.80 (1,475)	<0.0001
FEV1% (%)	80.4±6.38 (768)	81.7±6.6 (1,475)	<0.0001
Serum albumin (g/l)	44.5±3.0 (1,715)	44.7±2.4 (2,497)	0.0302
Serum total cholesterol (mmol/l)	5.19±0.90 (2,230)	5.23±0.88 (3,720)	0.0921
Serum triglyceride (mmol/l)	1.43±0.96 (2,215)	1.16±0.79 (3,721)	<0.0001
Serum HDL-cholesterol (mmol/l)	1.59±0.44 (2,213)	1.70±0.45 (3,721)	<0.0001
Serum LDL-cholesterol (mmol/l)	3.15±0.79 (2,212)	3.19±0.81 (3,720)	0.0632
Fasting plasma glucose (mmol/l)	5.90±1.36 (2,238)	5.40±0.96 (3,718)	<0.0001
Blood hemoglobin A1c (%)	5.84±0.78 (1,782)	5.59±0.59 (2,681)	<0.0001
Blood urea nitrogen (mmol/l)	5.72±2.68 (1,691)	4.86±1.23 (2,410)	<0.0001
Serum creatinine (*μ*mol/l)	88.5±127.4 (2,162)	64.8±15.1 (3,414)	<0.0001
eGFR (ml/min/1.73 m^−2^)	71.2±18.3 (2,162)	80.1±14.7 (3,414)	<0.0001
Serum uric acid (*μ*mol/l)	349±88 (2,139)	312±81 (3,392)	<0.0001
Serum C-reactive protein (*μ*g/l)	1832±9666 (775)	826±3359 (1,338)	0.0005
White blood cells (10^3^/*μ*l)	5.51±1.74 (1,573)	5.31±1.63 (3,034)	0.0001
Red blood cells (10^4^/*μ*l)	436±48 (1,577)	437±43 (3,046)	0.1928
Hemoglobin (g/l)	139±16 (1,577)	137±15 (3,046)	0.0017
Hematocrit (%)	40.4±4.4 (1,576)	40.1±4.2 (3,042)	0.0186
Platelets (10^4^/*μ*l)	21.8±5.5 (1,557)	22.6±5.3 (3,011)	<0.0001

a Values in parentheses indicate the numbers of measurements taken. Quantitative data are the means ± SD. eGFR, estimated glomerular filtration rate (ml/min/1.73 m^−2^) = 194 × [age (years)]^−0.287^ × [serum creatinine (mg/dl)]^−1.094^ × [0.739 if female]; HDL, high density lipoprotein; LDL, low density lipoprotein.

**Table V:** Association of polymorphisms with hypertension analyzed for 5-year longitudinal data with a generalized estimating equation.

Gene or locus	SNP	Genotype	Hypertension^a^	Controls^a^	P-value (dominant model)^b^	P-value (recessive model)^c^
*FAM78B*	rs2116519 (C→T)	*TT*	1,888 (32.3)	6,649 (30.3)	0.3039	**0.0266**
		*TC*	2,959 (50.7)	11,046 (50.3)		
		*CC*	991 (17.0)	4,279 (19.5)		
3q28	rs9846911 (A→G)	*AA*	5,033 (86.2)	19,102 (86.9)	0.1629	0.1620
		*AG*	759 (13.0)	2,756 (12.5)		
		*GG*	46 (0.8)	116 (0.5)		
*ALPK1*	rs2074379 (G→A)	*AA*	2,707 (46.4)	10,004 (45.5)	0.7330	0.2596
		*AG*	2,560 (43.9)	9,736 (44.3)		
		*GG*	571 (9.8)	2,234 (10.2)		
*ALPK1*	rs2074380 (G→A)	*GG*	4,905 (84.0)	18,656 (84.9)	0.1124	0.1496
		*GA*	885 (15.2)	3,165 (14.4)		
		*AA*	48 (0.8)	153 (0.7)		
*ALPK1*	rs2074381 (A→G)	*AA*	4,981 (85.3)	18,815 (85.6)	0.2390	0.4732
		*AG*	821 (14.1)	3,038 (13.8)		
		*GG*	36 (0.6)	121 (0.6)		
*ALPK1*	rs2074388 (G→A)	*AA*	2,714 (46.5)	10,013 (45.6)	0.7043	0.2637
		*AG*	2,552 (43.7)	9,721 (44.2)		
		*GG*	572 (9.8)	2,240 (10.2)		
*BTN2A1*	rs6929846 (T→C)	*CC*	4,484 (76.8)	17,333 (78.9)	**0.0013**	0.3602
		*CT*	1,275 (21.8)	4,365 (19.9)		
		*TT*	79 (1.4)	276 (1.3)		
*THBS2*	rs8089 (T→G)	*TT*	4,895 (83.8)	18,159 (82.6)	0.7407	0.9741
		*TG*	902 (15.5)	3,615 (16.5)		
		*GG*	41 (0.7)	200 (0.9)		
*PDX1*	rs146021107 (G→-)	*GG*	1,745 (29.9)	5,983 (27.2)	**0.0031**	0.2885
		*G/*-	2,839 (48.6)	11,049 (50.3)		
		-/-	1,254 (21.5)	4,942 (22.5)		
*F7*	rs6046 (G→A)	*GG*	5,104 (87.4)	19,187 (87.3)	0.1478	0.8979
		*GA*	715 (12.2)	2,693 (12.3)		
		*AA*	19 (0.3)	94 (0.4)		
*LLGL2*	rs1671021 (G→A)	*AA*	4,187 (71.7)	16,353 (74.4)	**0.0372**	0.3881
		*AG*	1,521 (26.1)	5,223 (23.8)		
		*GG*	130 (2.2)	398 (1.8)		
*ILF3*	rs2569512 (G→A)	*GG*	2,563 (43.9)	9,525 (43.3)	0.3765	0.2560
		*GA*	2,605 (44.6)	10,180 (46.3)		
		*AA*	670 (11.5)	2,269 (10.3)		
*CELSR1*	rs6007897 (C→T)	*TT*	5,671 (97.1)	21,303 (96.9)	0.6353	not determined
		*TC*	167 (2.9)	671 (3.1)		
		*CC*	0 (0)	0 (0)		

The prevalence of hypertension was compared between 2 groups (the dominant or recessive model) for each polymorphism with adjustment for age, gender, body mass index and smoking status.

a Values indicate the numbers of measurements taken, with the percentages in parentheses;

b dominant model: *AA* vs. *AB* + *BB* (*A*, major allele; *B*, minor allele);

c recessive model (*AA* + *AB* vs. *BB*). P-values of <0.05 are shown in bold. SNPs, single nucleotide polymorphisms.

**Table VI:** Association of polymorphisms with systolic, diastolic, or mean BP in all individuals or individuals not taking any anti-hypertensive medication analyzed for 5-year longitudinal data with a generalized linear mixed-effect model.

Gene	SNP	BP (mmHg)	Dominant model^a^		P-value	Recessive model^a^		P-value
All individuals								
*FAM78B*	rs2116519 (C→T)		*TT* (8,537)	*TC* + *CC* (19,275)		*TT* + *TC* (2,2542)	*CC* (5,270)	
		Systolic	121.0±16.7	120.4±16.1	0.3818	120.7±16.5	120.1±15.7	0.5823
		Diastolic	74.9±12.5	74.6±12.1	0.1260	74.8±12.4	74.1±11.8	0.0823
		Mean	90.2±13.1	89.9±12.6	0.1722	90.1±12.9	89.4±12.2	0.1814
*BTN2A1*	rs6929846 (T→C)		*CC* (21,817)	*CT* + *TT* (5,995)		*CC* + *CT* (27,457)	*TT* (355)	
		Systolic	120.4±16.2	121.2±16.7	**0.0061**	120.6±16.3	121.4±15.4	0.1369
		Diastolic	74.5±12.2	75.2±12.4	**0.0023**	74.7±12.3	75.2±11.0	0.2483
		Mean	89.8±12.7	90.5±13.0	**0.0019**	90.0±12.8	90.6±11.7	0.1748
*PDX1*	rs146021107 (G→-)		*GG* (7,728)	*G/*- + -/- (20,084)		*GG* + *G/*- (21,616)	-/- (6,196)	
		Systolic	121.1±17.1	120.4±16.0	**0.0284**	120.8±16.4	120.0±16.1	0.3884
		Diastolic	74.5±12.8	74.7±12.1	0.2719	74.8±12.3	74.4±12.1	0.9222
		Mean	90.1±13.3	90.0±12.5	0.1029	90.1±12.8	89.6±12.5	0.6821
*LLGL2*	rs1671021 (G→A)		*AA* (20,540)	*AG* + *GG* (7,272)		*AA* + *AG* (27,284)	*GG* (528)	
		Systolic	120.4±16.2	121.2±16.6	0.1943	120.6±16.3	121.6±16.1	0.9056
		Diastolic	74.5±12.2	75.2±12.4	0.1280	74.7±12.3	75.8±12.7	0.4665
		Mean	89.8±12.7	90.5±12.9	0.1315	90.0±12.8	91.1±12.8	0.7203
Individuals not taking any anti-hypertensive medication								
*FAM78B*	rs2116519 (C→T)		*TT* (8,132)	*TC* + *CC* (18,370)		*TT* + *TC* (2,1459)	*CC* (5,043)	
		Systolic	120.5±16.7	119.9±16.0	0.2563	120.2±16.4	119.7±15.6	0.5041
		Diastolic	74.6±12.5	74.4±12.1	0.2039	74.6±12.4	73.9±11.7	0.0495
		Mean	89.9±13.0	89.6±12.6	0.1948	89.8±12.9	89.1±12.1	0.1248
*BTN2A1*	rs6929846 (T→C)		*CC* (20,807)	*CT* + *TT* (5,695)		*CC* + *CT* (26,163)	*TT* (339)	
		Systolic	120.0±16.1	120.7±16.6	**0.0017**	120.1±16.3	120.8±15.3	0.1734
		Diastolic	74.3±12.2	75.0±12.4	**0.0008**	74.4±12.3	75.0±10.8	0.2059
		Mean	89.5±12.7	90.2±13.0	**0.0005**	89.7±12.7	90.3±11.5	0.1678
*PDX1*	rs146021107 (G→-)		*GG* (7,328)	*G/*- + -/- (19,174)		*GG* + *G/*- (20,580)	-/- (5,922)	
		Systolic	120.6±17.1	120.0±15.9	**0.0132**	120.3±16.3	119.5±16.0	0.2565
		Diastolic	74.2±12.8	74.5±12.0	0.3963	74.5±12.3	74.2±12.1	0.8832
		Mean	89.7±13.3	89.7±12.5	0.1081	89.8±12.8	89.3±12.5	0.7018
*LLGL2*	rs1671021 (G→A)		*AA* (19,569)	*AG* + *GG* (6,933)		*AA* + *AG* (26,005)	*GG* (497)	
		Systolic	119.9±16.2	120.7±16.5	0.0891	120.1±16.3	121.4±16.1	0.6847
		Diastolic	74.2±12.2	75.0±12.4	**0.0468**	74.4±12.2	75.7±12.7	0.2512
		Mean	89.5±12.7	90.2±12.9	**0.0471**	89.6±12.7	90.9±12.80	0.3889

Systolic, diastolic, or mean BP was compared between 2 groups (the dominant or recessive model) for each polymorphism with adjustment for age, gender, body mass index and smoking status.

a Values in parentheses indicate the numbers of measurements taken. Data for BP are the means ± SD. P-values of <0.05 are shown in bold. BP, blood pressure. SNPs, single nucleotide polymorphisms.
